# A CCHFV DNA vaccine protects against heterologous challenge and establishes GP38 as immunorelevant in mice

**DOI:** 10.1038/s41541-021-00293-9

**Published:** 2021-03-02

**Authors:** John J. Suschak, Joseph W. Golden, Collin J. Fitzpatrick, Charles J. Shoemaker, Catherine V. Badger, Connie S. Schmaljohn, Aura R. Garrison

**Affiliations:** 1 Virology Division, United States Army Medical Research Institute of Infectious Diseases, Fort Detrick, MD USA; 2 Diagnostics Systems Division, United States Army Medical Research Institute of Infectious Diseases, Fort Detrick, MD USA; 3 Headquarters Division, United States Army Medical Research Institute of Infectious Diseases, Fort Detrick, MD USA; 4 Present Address: National Institute of Allergy and Infectious Diseases, Integrated Research Facility, Frederick, MD USA

**Keywords:** Infectious diseases, Vaccines, Vaccines, Virology

## Abstract

Crimean-Congo hemorrhagic fever virus (CCHFV) is a tick-borne virus that causes severe hemorrhagic fever disease in humans. Currently, no licensed CCHF vaccines exist, and the protective epitopes remain unclear. Previously, we tested a DNA vaccine expressing the M-segment glycoprotein precursor gene of the laboratory CCHFV strain IbAr 10200 (CCHFV-M_10200_). CCHFV-M_10200_ provided >60% protection against homologous CCHFV-IbAr 10200 challenge in mice. Here, we report that increasing the dose of CCHFV-M_10200_ provides complete protection from homologous CCHFV challenge in mice, and significant (80%) protection from challenge with the clinically relevant heterologous strain CCHFV-Afg09-2990. We also report complete protection from CCHFV-Afg09-2990 challenge following vaccination with a CCHFV-Afg09-2990 M-segment DNA vaccine (CCHFV-M_Afg09_). Finally, we show that the non-structural M-segment protein, GP38, influences CCHF vaccine immunogenicity and provides significant protection from homologous CCHFV challenge. Our results demonstrate that M-segment DNA vaccines elicit protective CCHF immunity and further illustrate the immunorelevance of GP38.

## Introduction

Crimean-Congo hemorrhagic fever virus (CCHFV) is a tick-borne member of the family *Nairoviridae* in the order *Bunyavirales* with the widest geographical distribution. CCHFV infection in humans causes a severe and often fatal disease with a mortality rate ranging from 3-60%^1^. CCHFV has a tripartite, negative sense RNA genome comprised of a small (S), medium (M), and large (L) segment. The S segment encodes the nucleocapsid (N) protein, the M-segment encodes the glycoprotein precursor complex (GPC), containing two glycoproteins (G_N_ and G_C_) as well as several non-structural proteins (mucin-like domain, GP38, GP160, GP85, and NS_M_), and the L segment encodes the RNA-dependent RNA polymerase^2^. Depending on the algorithm used in analysis, CCHFV is the most genetically diverse of the arboviruses with 6–7 virus clades, and with nucleotide divergence of 20% among the S-segments, 22% among the L-segments, and the largest divergence of 31% among the M segments^1^. Although the M-segment is the most diverse, the majority of the variation is in the N-terminal non-structural domains. The optimal CCHF vaccine will account for this high genetic diversity and confer broad protection against divergent strains. As of 2015, CCHF is designated a top ten priority emerging infectious disease by the World Health Organization^3,4^. This classification has led to increased focus on the development of a protective CCHF vaccine.

CCHF vaccine efforts are hindered by a lack of knowledge regarding the necessary immune response(s) required for protection against disease. While robust levels of IgM and IgG have been identified as clinical indicators of survival^5^, the correlates of protection remain to be established experimentally^6^. In mouse models, antibody responses following vaccination appear beneficial, however, they do not necessarily predict survival^6–8^, and a correlation between the humoral response and survival has not been established^7–10^. Furthermore, adoptive transfer of T cells or sera from vaccinated mice to naïve recipients does not significantly improve survival following CCHFV challenge, but a combination of anti-CCHFV T cells and antibodies offers some protection^11^. These data suggest that both arms of the adaptive immune response are critical for controlling CCHFV infection, and that any efficacious vaccine will need to elicit antigen-specific T cells and antibodies.

Most CCHF vaccines focus on the M-segment, as the glycoproteins are accessible on the virion surface and are the target of neutralizing and non-neutralizing antibodies. Multiple vaccine platforms containing either the full-length M-segment or G_N_/G_C_ have been described^7,9,12^. Notably, DNA vaccination has proven capable of eliciting protective anti-CCHFV immune responses, possibly due to the ability of DNA vaccines to generate both humoral and cellular immunity. A triple plasmid formulation consisting of individual plasmids expressing G_N_, G_C_, and N yielded high-level antibody responses, and conferred protection against CCHFV challenge^10^, but the protective response elicited by each vaccine component was not fully characterized, and N vaccination alone can offer significant protection^13,14^.

We previously reported the use of a single plasmid DNA vaccine expressing the codon-optimized full-length CCHFV M-segment (CCHFV-M_10200_) open reading frame (ORF)^8^. We also reported on a transiently immune-suppressed (IS) C57BL/6 mouse model wherein type I interferon (IFN-I) signaling is blocked by monoclonal antibody (mAb)-5A3 treatment proximal to challenge. As IFN-I signaling is only blocked during the challenge, this model allows for the development of potent vaccine-elicited immune responses, while still providing a lethal CCHFV murine model for testing vaccine efficacy^8^. In our initial study, three doses of 25 μg of CCHFV-M_10200_ delivered by intramuscular (IM) electroporation (EP) provided >60% protection from lethal CCHFV infection in both the type I interferon receptor (IFNAR^-/-^) and the IS models. The two mouse models had comparable humoral immune responses, except the IS mice had a more balanced IgG2a/IgG1 response to vaccination. In agreement with previous reports^7,9^, our data indicated no direct correlation between the humoral response and survival in CCHFV-M_10200_ vaccinated mice, as even some mice that succumbed to disease developed high levels of neutralizing antibodies.

The majority of CCHF vaccines have been tested against homologous challenge. Canakoglu et al. tested a formalin-inactivated vaccine based on the Turkey-Kelkit06 strain^15^, but the majority of CCHF vaccines, including ours, are designed using the laboratory-adapted strain, CCHFV-IbAr 10200. Two notable exceptions of heterologous challenge studies are a virus-like replicon particle (VRP) vaccine expressing the GPC of the Oman-1998 strain tested against CCHFV-IbAr 10200, and a replication-competent recombinant vesicular stomatitis virus (rVSV) expressing the CCHFV-IbAr 10200 GPC against the Turkey 200406546 strain^12,16^. It remains to be fully investigated if CCHFV-IbAr 10200-based vaccines can provide optimal cross protection against challenge with other clinically relevant CCHFV strains. Additionally, the divergent epitopes that may influence cross-protection require examination.

In this report, we further expanded on our efforts to produce a simplified, efficacious CCHFV M-segment vaccine. We tested an increased vaccine dose in an effort to improve the immune response and provide complete protection against lethal CCHFV challenge with the homologous strain, as well as provide cross-protection against a divergent CCHFV strain. We also designed and tested a DNA vaccine expressing the M-segment of the clinically relevant CCHFV-Afg09-2990 strain. Finally, we investigated the role of the non-structural M-segment protein, GP38, to gain a clearer understanding of its role in CCHF vaccine efficacy.

## Results

### CCHFV-M_10200_ protects mice from homologous challenge

**Fig. 1:**
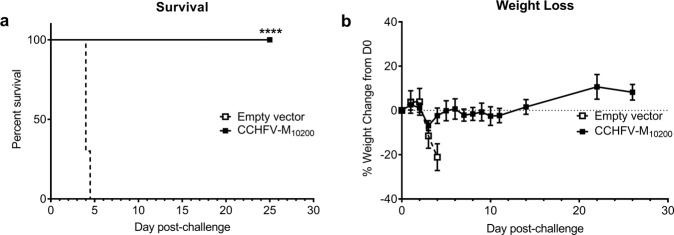
CCHFV-M_10200_ DNA vaccination completely protects against homologous challenge. Groups of 10 C57BL/6 mice were vaccinated with 50 μg CCHFV-M_10200_ or empty vector on days 0, 21, and 42 by IM-EP. **a** Group survival and (**b**) weight change of CCHFV-M_10200_ vaccinated mice following CCHFV challenge with 100 PFU by the IP route with the homologous CCHFV-IbAr 10200 strain. Vaccinated C57BL/6 mice were transiently immunosuppressed prior to challenge. ****p < 0.0001. The percent survival *p* value was determined by a log rank test.

In earlier studies, we demonstrated partial protection against homologous CCHFV challenge in mice vaccinated with CCHFV-M_10200_. To test the protective efficacy of an increased dose of our CCHFV-M_10200_ vaccine, groups of 10 C57BL/6 mice were vaccinated three times, three weeks apart, with 50 µg of our CCHFV-M_10200_ DNA vaccine or an empty vector control by IM-EP as described in the Methods. Four weeks following the final vaccination, the mice were treated with mAb-5A3 to block IFN-I signaling and challenged via the intraperitoneal (IP) route with 100 plaque forming units (PFU) of the homologous laboratory strain, CCHFV-IbAr 10200. The CCHFV-M_10200_ vaccine provided 100% protection against CCHFV-IbAr 10200 challenge (Fig. 1a), with no signs of illness (lethargy, ruffling). Conversely, all empty vector controls developed visible signs of illness and succumbed to infection or reached euthanasia criteria by 4.5 days post-infection. The CCHFV-M_10200_ vaccinated group had minimal transient weight loss in comparison to the empty vector control group (Fig. 1b).

### The CCHFV-M_10200_ DNA vaccine elicits antigen-specific cellular and humoral immunity

**Fig. 2:**
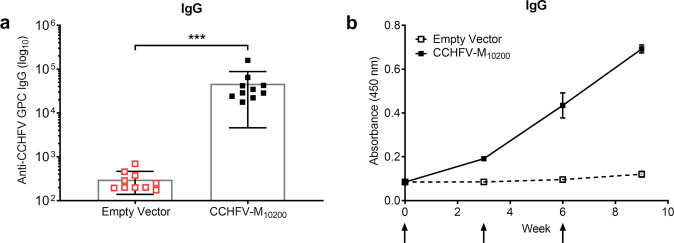
CCHFV-M_10200_ DNA vaccination elicits CCHFV-IbAr 10200 specific antibodies. Groups of 10 female C57BL/6 mice were vaccinated with 50 μg CCHFV-M_10200_ or empty vector on days 0, 21, and 42 by IM-EP. **a** CCHFV-IbAr 10200-specific antibody responses were analyzed 21 days post third vaccination for ELISA endpoint titers. Mice that succumbed to challenge are highlighted red. **b** Temporal anti-CCHFV-IbAr 10200 IgG responses in vaccinated mice. The arrows indicate DNA vaccination time points. Data are the group mean averages ± SD. ***p < 0.001. The endpoint titer *p* value was determined by Student’s *t* test with a 95% confidence interval.

As our CCHFV-M_10200_ vaccine conferred complete protection against viral challenge, we investigated the possible immune correlates of protection. We initially quantified the antibody response elicited by our CCHFV-M_10200_ DNA vaccine. For this analysis, we utilized lysates from CCHFV-IbAr 10200 CCHF_VLP_ transfected cells. The choice to use cell lysates instead of secreted CCHF_VLP_, as previously published^8^, was to capture the antibody responses to both the structural and non-structural proteins. IM-EP delivery of the CCHFV-M_10200_ vaccine yielded high-level, CCHFV GPC-specific antibody titers (Fig. 2a) that were boosted by each subsequent vaccination (Fig. 2b). These data suggest that at a minimum, three vaccinations are required to reach the peak antibody response. A comparison of CCHFV GPC-specific antibody titers quantified by either secreted CCHF_VLP_ or CCHFV-IbAr 10200 CCHF_VLP_ transfected cell lysates is presented in Supplementary Fig. 1.

**Fig. 3:**
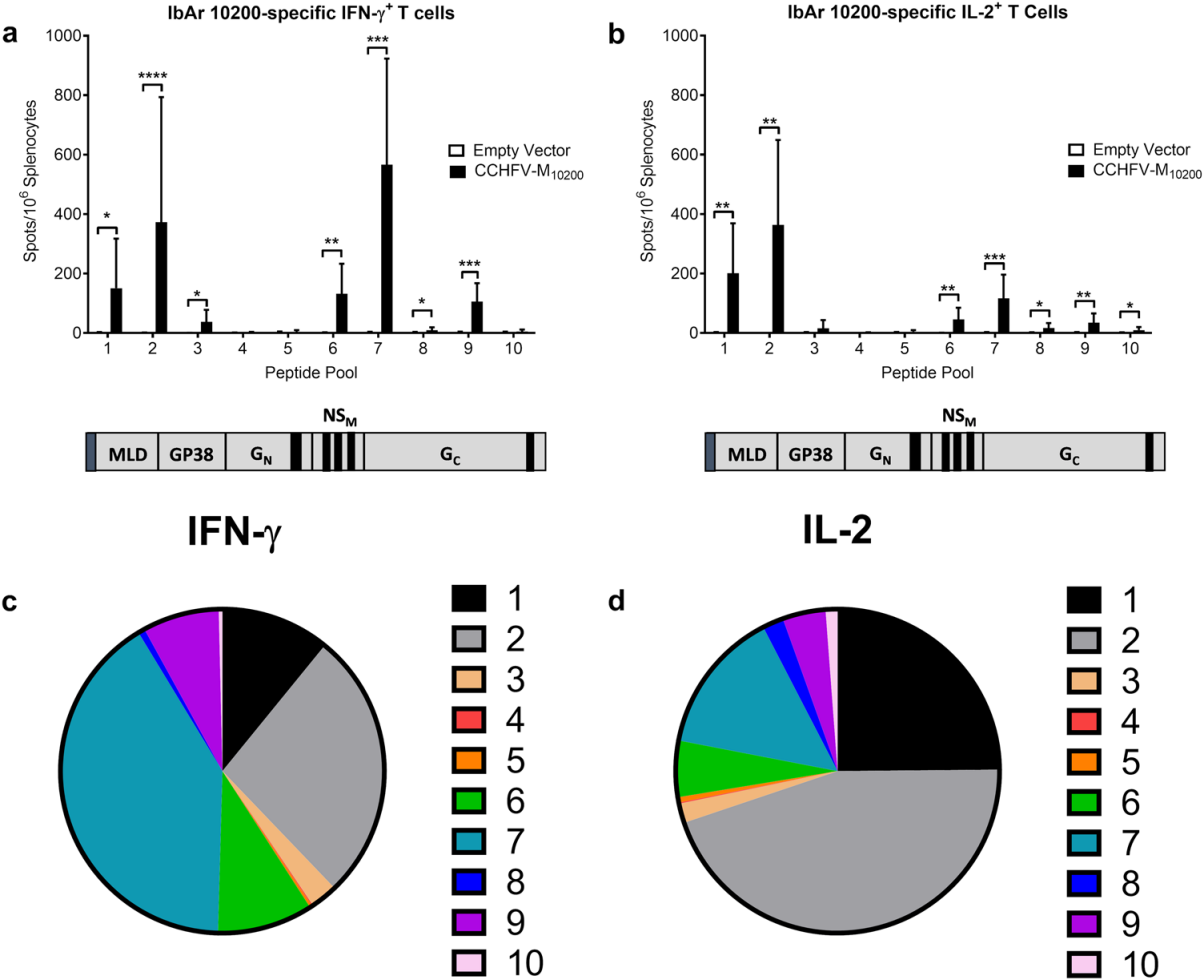
Anti-M cellular immune responses are specific to defined regions. Groups of 9 female C57BL/6 mice were vaccinated with 50 μg CCHFV-M_10200_ or empty vector on days 0, 21, and 42 by IM-EP, and then euthanized on day 49 for splenocyte T cell analysis. Splenocytes from individual mice were re-stimulated with pooled peptides derived from the CCHFV-IbAr 10200 M-segment. Anti-CCHFV-M specific (**a**) IFN-γ^+^ and (**b**) IL-2^+^ T cells were quantified by ELISPOT. The corresponding region of the peptide pools to the M-segment is shown below each graph. Percentage of (**c**) IFN-γ^+^ and (**d**) IL-2^+^ T cells responding to each peptide pool. Data are the group mean averages ± SD. *p < 0.05; **p < 0.01; ***p < 0.001; ***p < 0.0001. *p* values were determined by two-way ANOVA with Sidak’s multiple comparison test with a 95% confidence interval.

To examine the T-cell response elicited by the CCHFV-M_10200_ DNA vaccine, we vaccinated a cohort of mice (*n* = 9) three times as above, and one week following the final vaccination splenocytes were isolated for IFN-γ^+^ and IL-2^+^ T cell ELISPOT. Splenocytes were stimulated with ten individual peptide pools spanning the length of the CCHFV-IbAr 10200 M-segment open reading frame. The majority of IFN-γ^+^ (Fig. 3a, c) and IL-2^+^ (Fig. 3b, d) T-cell responses were mapped to the mucin-like domain (MLD) and GP38 non-structural proteins, as well as to NS_M_ and the N-terminus of G_C_. Peptide pools spanning G_N_ did not stimulate an antigen-specific T cell response. We also quantified cytokine signaling in CCHFV-M_10200_ vaccinated mice from sera harvested at the time of euthanasia. CCHFV-M_10200_ vaccinated mice had significant increases in the T_h_1 cytokines TNF-α and IL-12, and the T_h_2 cytokine IL-6 after three vaccinations (Supplementary Fig. 2). These results further demonstrate the ability of DNA vaccines to elicit balanced adaptive immune responses.

### Vaccination with the CCHFV-M_10200_ DNA vaccine generates cross-clade immunity, and is partially protective against heterologous challenge

**Fig. 4:**
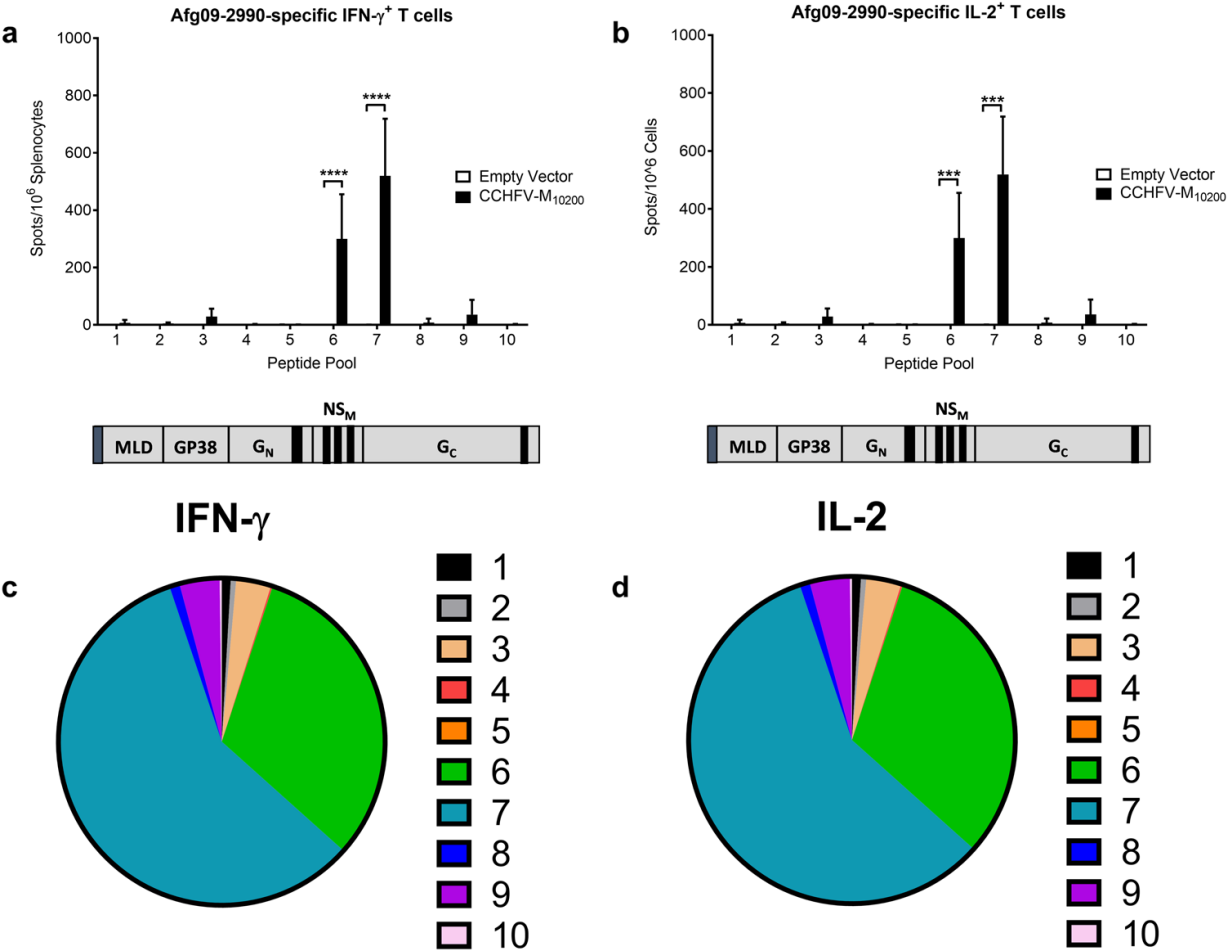
CCHFV-IbAr 10200-specific T cells are not cross-reactive with the CCHFV-Afg09-2990 MLD or GP38. Groups of 10 female C57BL/6 mice were vaccinated with 50 μg CCHFV-M_10200_ or empty vector on days 0, 21, and 42 by IM-EP, and then euthanized on day 49 for splenocyte T cell analysis. Splenocytes from individual mice were re-stimulated with pooled peptides derived from the CCHFV strain Afg09-2990 M-segment. Anti-CCHFV-Afg09-2990 specific (**a**) IFN-γ^+^ and (**b**) IL-2^+^ T cells were quantified by ELISPOT. The corresponding region of the peptide pools to the M-segment are shown below each graph. Percentage of (**c**) IFN-γ^+^ and (**d**) IL-2^+^ T cells responding to each peptide pool. Data are the group mean averages ± SD. ***p < 0.001; ****p < 0.0001. *p* values were determined by two-way ANOVA with Sidak’s multiple comparison test with a 95% confidence interval.

Since our CCHFV-IbAr 10200 vaccine was completely protective against homologous challenge, we tested the cross-protective efficacy of the CCHFV-M_10200_ DNA vaccine against a clinically relevant strain, CCHFV-Afg09-2990, from a divergent clade of CCHFV strains. Groups of 20 C57BL/6 mice were vaccinated with 50 μg of our CCHFV-M_10200_ DNA vaccine, three times, three weeks apart, or an empty vector control by IM-EP as described in the Methods. One week after the final vaccination, 10 mice from each group were euthanized for T cell ELISPOT analysis. Splenocytes were stimulated with peptide pools spanning the CCHFV-Afg09-2990 M-segment open-reading frame, and the IFN-γ^+^ (Fig. 4a, c) and IL-2^+^ (Fig. 4b, d) T cell responses were quantified. Surprisingly, the pools spanning the MLD of CCHFV-Afg09-2990 did not elicit a T-cell response in CCHFV-M_10200_ vaccinated mice, and one pool (Pool #3) spanning GP38 elicited only a minimal response. However, CCHFV-M_10200_ vaccination elicited potent T cell responses directed against the CCHFV-Afg09-2990 pools spanning the NS_M_ and N-terminal domain of G_C_, similar to the response seen in splenocytes stimulated with the homologous CCHFV-IbAr 10200 peptides.

**Fig. 5:**
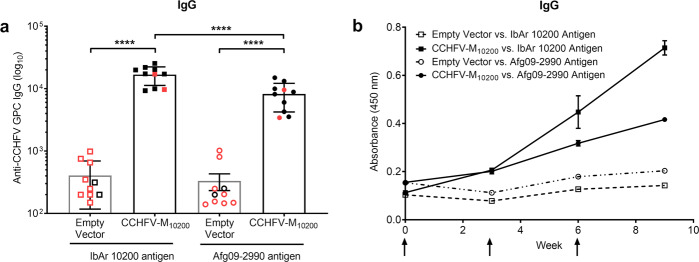
CCHFV-M_10200_ DNA vaccination elicits antibodies that are partially cross-reactive to CCHFV-Afg09-2990. Groups of 10 female C57BL/6 mice were vaccinated with 50 μg CCHFV-M_10200_ or empty vector on days 0, 21, and 42 by IM-EP. **a** Sera antibody responses were analyzed 21 days post third vaccination for anti-CCHFV-IbAr 10200 or anti-CCHFV-Afg09-2990 endpoint titers by ELISA. Mice that succumbed to viral challenge are shown in red. **b** Temporal anti-CCHFV IgG responses in vaccinated mice. The arrows indicate DNA vaccination time points. Data are the group mean averages ± SD. ****p < 0.0001. *p* values were determined by one-way ANOVA with Tukey’s post hoc test with a 95% confidence interval.

We then followed the remaining mice (*n* = 10) for an additional two weeks to assess the development of anti-CCHFV antibodies. For this analysis, we tested sera from CCHFV-M_10200_ vaccinated mice against not only CCHFV-IbAr 10200 antigen, but also CCHFV-Afg09-2990 antigen. All CCHFV-M_10200_ vaccinated mice generated antibodies as observed in the initial homologous viral challenge study. CCHFV-M_10200_ vaccination elicited anti-CCHFV-IbAr 10200 antibody titers that were comparable to those measured in the previous study, but we observed a significant decrease in antibody titers when the same sera were tested against the CCHFV-Afg09-2990 antigen (Fig. 5a). Although anti-CCHFV-Afg09-2990 antibody levels were boosted after each vaccination, they failed to reach the overall level measured against CCHFV-IbAr 10200 antigen, suggesting at least a partial divergence in humoral epitopes (Fig. 5b).

**Fig. 6:**
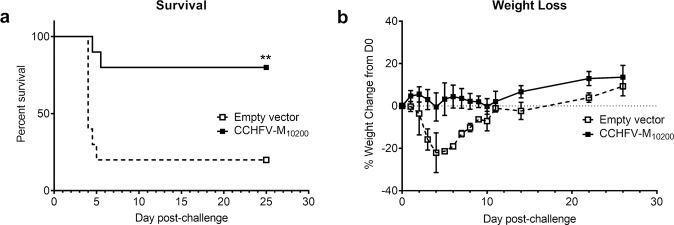
CCHFV-M_10200_ DNA vaccination provides significant protection against heterologous challenge. Groups of 10 female C57BL/6 mice were vaccinated with 50 μg CCHFV-M_10200_ or empty vector on days 0, 21, and 42 by IM-EP. **a** Group survival and (**b**) weight change following CCHFV challenge with 100 PFU by the IP route with the heterologous CCHFV-Afg09-2990 strain. Vaccinated C57BL/6 mice were transiently immunosuppressed prior to challenge. **p < 0.01. The percent survival *p* value was determined by a log rank test.

Four weeks after the final vaccination, all remaining mice were treated by IP injection with mAb-5A3 and challenged by IP injection of 100 PFU CCHFV-Afg09-2990. Two mice in the empty vector group survived challenge with strain CCHFV-Afg09-2990 (Fig. 6a); however, these mice had significant weight loss and a ruffled appearance (Fig. 6b). In contrast, the CCHFV-M_10200_ DNA vaccine provided 80% protection against CCHFV strain CCHFV-Afg09-2990 (Fig. 6a). The two vaccinated mice that succumbed to CCHFV challenge had a delay in weight loss compared to the vector only control mice, and did not display other signs of illness such as ruffled fur (Fig. 6b). As was noted in our previous report^8^, there was no correlation between antibody titer and protection from CCHFV challenge.

### A DNA vaccine expressing the CCHFV-Afg09-2990 M-segment completely protects against CCHFV-Afg09-2990 challenge

**Fig. 7:**
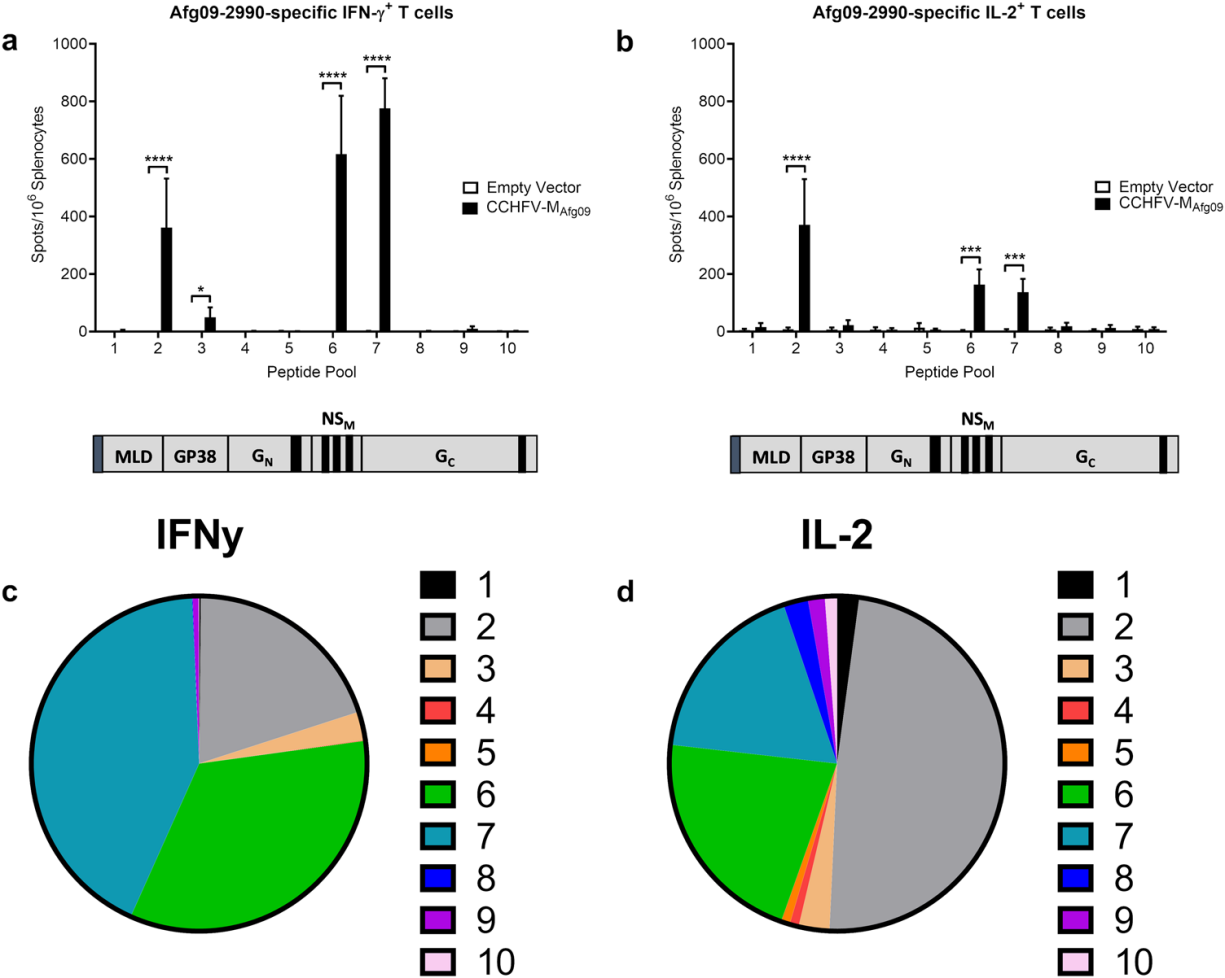
Anti-GP38 cellular immune responses are rescued following homologous CCHFV-M_Afg09_ vaccination. Groups of 10 female C57BL/6 mice were vaccinated with 50 μg CCHFV-M_Afg09_ or empty vector on days 0, 21, and 42 by IM-EP, and then euthanized on day 49 for splenocyte T cell analysis. Splenocytes from individual mice were re-stimulated with pooled peptides derived from the CCHFV-Afg09-2990 M-segment. Anti-CCHFV-M specific (**a**) IFN-γ^+^ and (**b**) IL-2^+^ T cells were quantified by ELISPOT. The corresponding region of the peptide pools to the M-segment are shown below each graph. Percentage of (**c**) IFN-γ^+^ and (**d**) IL-2^+^ T cells responding to each peptide pool. Data are the group mean averages ± SD. *p < 0.05; ***p < 0.001; ****p < 0.0001. *p* values were determined by two-way ANOVA with Sidak’s multiple comparison test with a 95% confidence interval.

In an effort to improve upon the partial protection we measured in CCHFV-M_10200_ vaccinated mice challenged with CCHFV-Afg09-2990, we generated a DNA vaccine expressing the M-segment from CCHFV-Afg09-2990 (CCHFV-M_Afg09_) (Supplementary Fig. 3). Groups of 20 C57BL/6 mice were vaccinated with 50 μg of CCHFV-M_Afg09_ DNA vaccine or empty vector control by IM-EP as described in the Methods. As above, 10 mice from each group were euthanized for T cell ELISPOT analysis one week post final vaccination. Splenocytes were stimulated with peptide pools spanning the CCHFV-Afg09-2990 M-segment open-reading frame, and the IFN-γ^+^ (Fig. 7a, c) and IL-2^+^ (Fig. 7b, d) T cell responses were quantified. Whereas CCHFV-M_10200_ vaccinated mice only generated a T cell response to the CCHFV-Afg09-2990 G_C_ segment, mice vaccinated with CCHFV-M_Afg09_ developed significant levels of MLD, GP38, and G_C_-specific T cells. Unexpectedly, CCHFV-M_Afg09_ vaccination did not elicit a T cell response against CCHFV-Afg09-2990 MLD peptide Pool 1 as was measured in CCHFV-M_10200_ vaccinated mice. These data suggest that elicitation of cellular immune responses against the GP38 domain may require homologous vaccination.

**Fig. 8:**
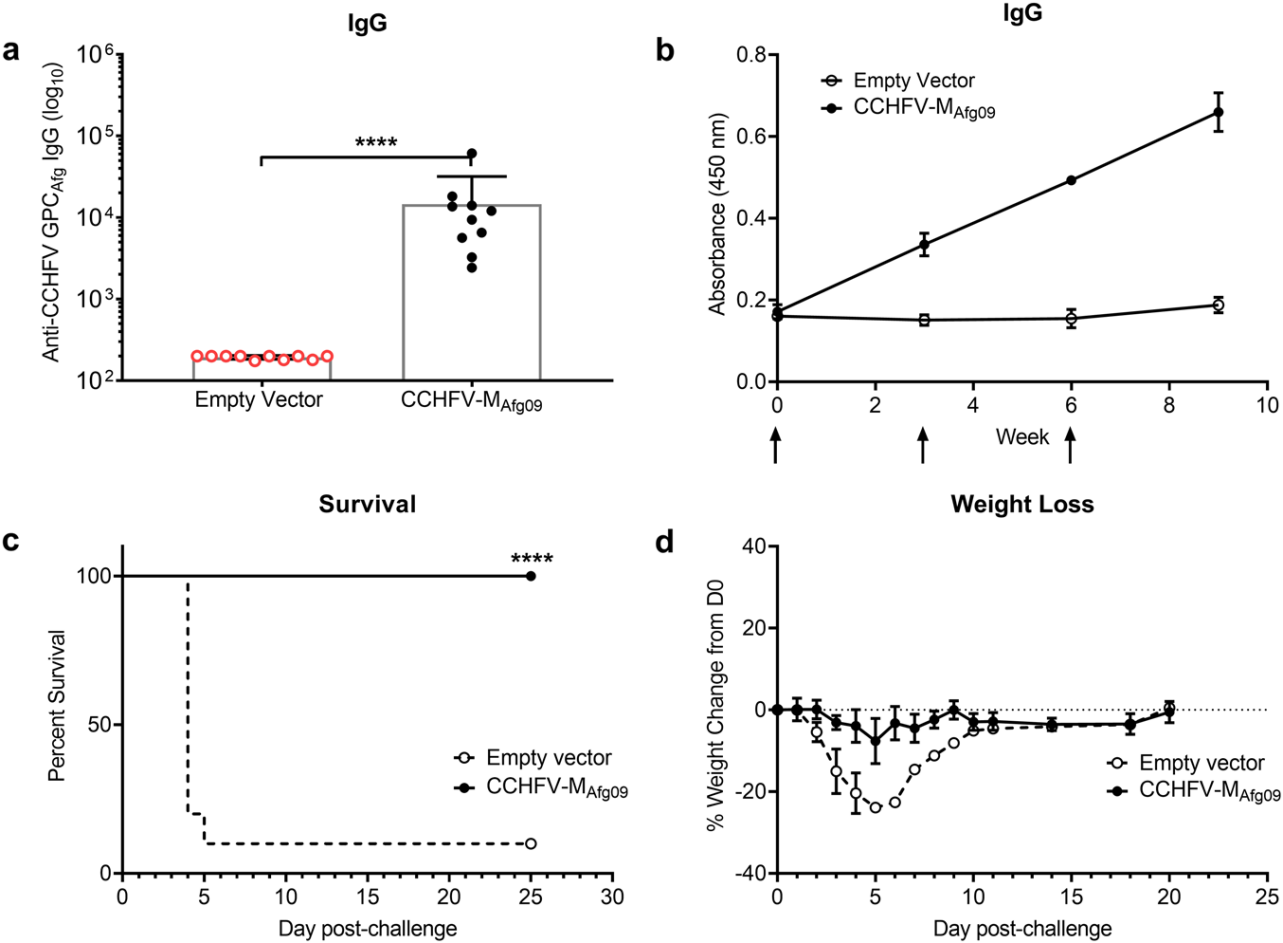
CCHFV-M_Afg09_ DNA vaccination is completely protective against homologous challenge. Groups of 10 female C57BL/6 mice were vaccinated with 50 μg CCHFV-M_Afg09_ or empty vector on days 0, 21, and 42 by IM-EP. **a** Sera antibody responses were analyzed 21 days post third vaccination for anti-CCHFV-Afg09-2990 endpoint titers by ELISA. Mice that succumbed to viral challenge are shown in red. **b** Temporal anti-CCHFV-Afg09-2990 IgG responses in vaccinated mice. The arrows indicate DNA vaccination time points. **c** Group survival and (**d**) weight change following CCHFV challenge with 100 PFU by the IP route with CCHFV-Afg09-2990. Vaccinated C57BL/6 mice were transiently immunosuppressed prior to challenge. Data are the group mean averages ± SD. ****p < 0.0001. The endpoint titer *p* value was determined by Student’s *t* test with a 95% confidence interval. The *p* value for percent survival was determined by log rank test with a 95% confidence interval.

We continued to follow the remaining mice (*n* = 10) for an additional two weeks to assess the development of anti-CCHFV-Afg09-2990 GPC antibodies. All CCHFV-M_Afg09_ vaccinated mice seroconverted while empty vector controls failed to develop CCHFV-Afg09-2990 GPC-specific antibodies (Fig. 8a, b). Four weeks after the final vaccination, all remaining mice were treated by IP injection of mAb-5A3 and challenged by 100 PFU of CCHFV-Afg09-2990. One mouse in the empty vector group survived challenge (Fig. 8c); however, this mouse exhibited approximately 24% weight loss and a ruffled appearance (Fig. 8d). In contrast, CCHFV-M_Afg09_ completely protected all mice from challenge (Fig. 8c) with only transient weight loss (Fig. 8d).

### GP38 DNA vaccination partially protects against CCHFV challenge

**Fig. 9:**
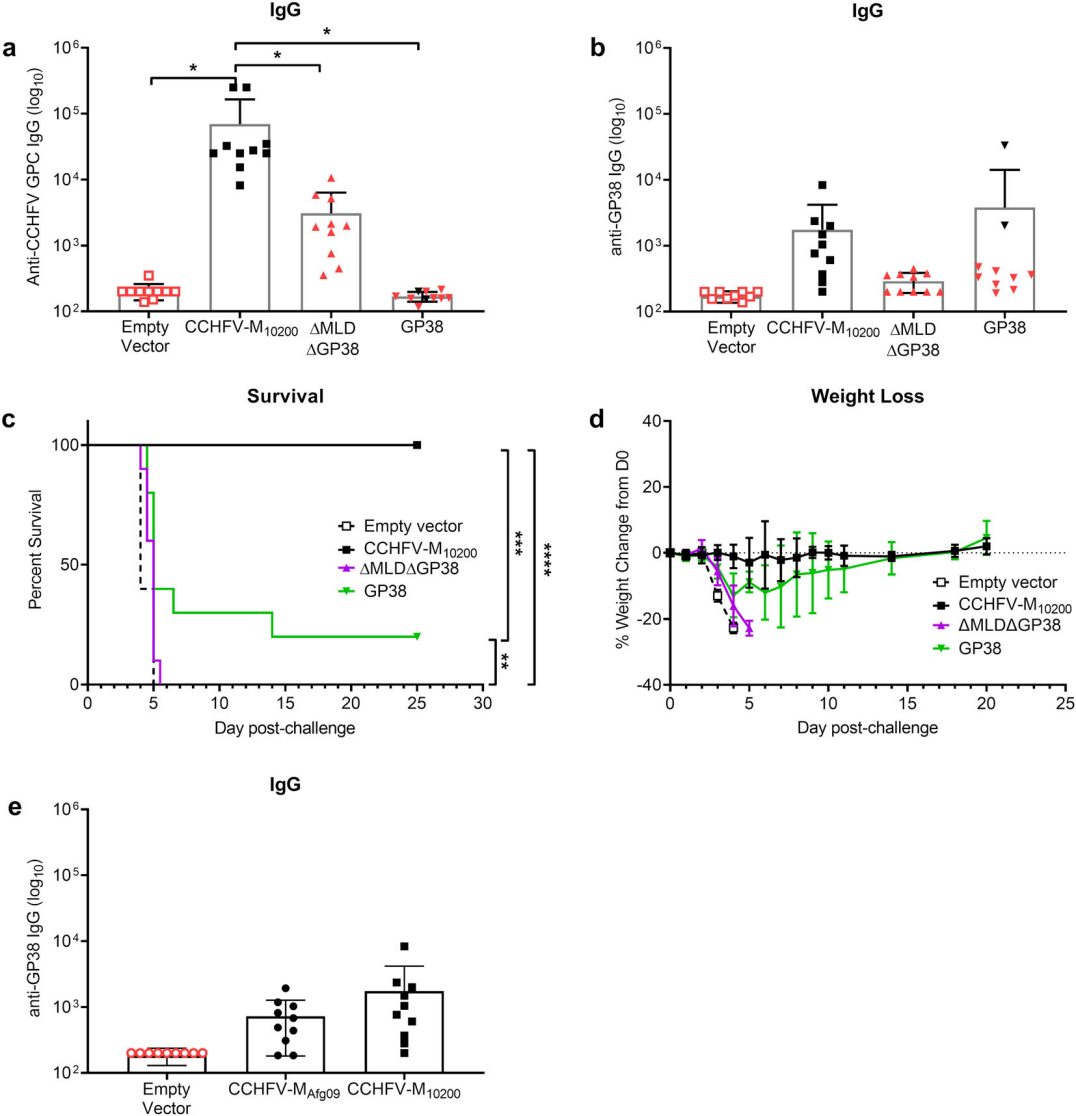
GP38 vaccination is partially protective against homologous challenge. Groups of 10 female C57BL/6 mice were vaccinated with 50 μg empty vector, CCHFV-M_10200_, ΔMLDΔGP38, or GP38 on days 0, 21, and 42 by IM-EP. **a** Sera antibody responses were analyzed 21 days post third vaccination for anti-CCHFV endpoint titers by ELISA. Mice that succumbed to viral challenge are shown in red. **b** Anti-CCHFV-IbAr 10200 GP38 IgG responses in vaccinated mice. **c** Group survival and (**d**) weight change following challenge with 100 PFU by the IP route with CCHFV-IbAr 10200. Vaccinated C57BL/6 mice were transiently immunosuppressed prior to challenge. **e** Anti-CCHFV-IbAr 10200 GP38 IgG ELISA titers in mice vaccinated with 50 μg empty vector, CCHFV-M_10200_, or CCHFV-M_Afg09_. Data are the group mean averages ± SD. *p < 0.05; **p < 0.01; ***p < 0.001; ****p < 0.0001. Endpoint titer *p* values were determined by one-way ANOVA with Tukey’s post hoc test with a 95% confidence interval. *p* values for percent survival were determined by log rank test with a 95% confidence interval.

As we found the most significant changes in immune profile between the CCHFV-M_10200_ and CCHFV-M_Afg09_ vaccine in the MLD and the GP38 regions, we hypothesized that a DNA vaccine expressing the GP38 region of CCHFV-IbAr 10200 would confer protection against homologous challenge. We therefore tested our previously described plasmids expressing either CCHFV-IbAr 10200 GP38, or a truncated CCHFV-IbAr 10200 M-segment with deleted MLD and GP38 regions (ΔMLDΔGP38) as DNA vaccines (Supplementary Fig. 4)^17^. Groups of mice (*n* = 10) were vaccinated three times with 50 µg of CCHFV-M_10200_, GP38, or ΔMLDΔGP38 DNA vaccines by IM-EP. A control group of mice received 50 µg of empty vector. Three weeks after the final vaccination, we quantified both total anti-CCHFV-IbAr 10200 GPC (Fig. 9a) and anti-CCHFV-IbAr 10200 GP38-specific (Fig. 9b) IgG. Vaccination with full-length CCHFV-M_10200_ elicited antibodies to both GPC and GP38 proteins. ΔMLDΔGP38 vaccinated mice had anti-CCHFV GPC antibodies, but did not have significant levels of antibodies directed against GP38 as expected. GP38 vaccinated mice exhibited similar levels of anti-GP38-specific antibodies to the CCHFV-M_10200_ group.

Four weeks after the final vaccination, all mice were treated by IP injection of mAb-5A3 and challenged by IP injection of 100 PFU of CCHFV-IbAr 10200. All mice in the empty vector group became moribund and succumbed to infection or were euthanized (Fig. 9c). All CCHFV-M_10200_ vaccinated mice survived the challenge and maintained weight throughout the study (Fig. 9d). Two GP38 vaccinated mice survived viral challenge without developing overt signs of disease, and a third had a delayed time to death. The two surviving mice had the highest anti-GP38 antibody titers. Surprisingly, all ΔMLDΔGP38 vaccinated mice succumbed to infection at an equivalent rate to the empty vector group. These data suggest that high levels of anti-GP38 antibodies can provide protection from CCHFV challenge, while G_N_/G_C_ epitopes alone do not elicit sufficient immunity for protection.

Finally, because we had previously observed that T cells from CCHFV-M_10200_ vaccinated mice did not respond to CCHFV-Afg09-2990 GP38 peptide stimulation, we compared the anti-CCHFV-IbAr 10200 GP38 antibody response from CCHFV-M_10200_ and CCHFV-M_Afg09_ vaccinated mice (Fig. 9e). Anti-GP38 titers in CCHFV-M_Afg09_ vaccinated mice trended approximately two fold lower than did titers measured in CCHFV-M_10200_ vaccinated mice, suggesting that the humoral response directed against GP38 is at least partially strain specific.

## Discussion

DNA vaccines elicit high levels of humoral and cellular immunity, making DNA vaccination an ideal platform for combatting emerging infectious diseases^18^. This is especially important in instances when the correlate of protection remains unknown, as is the case with CCHF. It was previously established that DNA vaccines can completely protect mice from homologous CCHFV challenge^10^. That vaccine consisted of three separate plasmids (G_N_, G_C_, N) delivered as a mixed formulation. Here we report single plasmid vaccines expressing full-length M-segments of two diverse CCHFV strains. This approach provides three key benefits: (1) it ensures that all transfected cells receive the necessary components to express structurally relevant CCHFV glycoprotein, (2) it generates immunity to multiple M-segment epitopes, and (3) it streamlines vaccine production and scale up.

Our original study showed that a 25 µg dose of a DNA vaccine expressing only the M-segment (CCHFV-M_10200_) of the prototypic laboratory CCHFV-IbAr 10200 conferred 60% protection from homologous challenge^8^. Here we tested a 50 µg dose of CCHF-M_10200_, and also developed a vaccine based on the clinically relevant CCHFV-Afg09-2990 strain (CCHFV-M_Afg09_). Both the CCHFV-M_10200_ and CCHFV-M_Afg09_ vaccines were highly immunogenic, eliciting significant anti-CCHFV GPC humoral and cellular immune responses, and completely protected mice against their respective homologous challenges. These results provide further evidence that the CCHFV M-segment alone is sufficient for balanced, protective immunity^8,9,12^. The roles of cellular and humoral immunity in protection from CCHFV challenge remains unclear, as we observed no direct correlation between anti-CCHFV GPC IgG titers or anti-GPC-specific T cell populations and survival in our heterologous challenge experiments. It is probable that both anti-GPC T and B cell responses are required for protection. Of note, several vaccines have included N in their formulation^10,13^, but the absence of N in our vaccines did not negatively impact protective efficacy, as all mice survived homologous challenge.

The protective targets for a CCHF vaccine remain unclear, but our results may provide some insight into the role defined M-segment antigenic regions play. It was reported that neutralizing and non-neutralizing antibodies directed against the unprocessed G_N_ complex or the G_C_ region of the glycoprotein can protect neonatal mice from CCHFV challenge, suggesting their potential as antibody targets^19^. We therefore predicted that our ΔMLDΔGP38 vaccine would confer some level of protection from challenge. A previous attempt to generate protective immunity with either G_N_ or G_C_ recombinant protein was unsuccessful^7^, but we expected that plasmid vaccination would prove beneficial as expressing both proteins results in stable cell surface glycoprotein expression^8,19^. However, there was no significant difference in time-to-death between the ΔMLDΔGP38 and empty vector groups. Our findings reflect the lack of in vivo protection in adult mice with antibodies targeting G_C_^10,17^. Why vaccination with G_N_/G_C_ alone does not confer protection remains unknown, especially as we measured potent anti-G_C_ T cell responses in both CCHFV-M_10200_ and CCHFV-M_Afg09_ vaccinated mice and epitope mapping from clinical samples showed strong G_N_ and G_C_ reactivity^20^. These data imply that undefined epitopes within the M-segment may be critical for survival.

So what M-segment regions contribute to vaccine efficacy? The answer may partially lie in GP38. We previously reported that pre-challenge treatment with mAb-13G8, a non-neutralizing monoclonal antibody directed against CCHFV-IbAr 10200 GP38, protected against homologous CCHFV challenge in mice^17^. Here, we demonstrated partial, but significant, protection with a GP38-only vaccine. Mapping studies of B cell epitopes have identified the MLD and GP38 as key epitopic regions^10,20^. We hypothesize that GP38 has a broad impact on protective efficacy in a strain-specific manner. This hypothesis is supported by the partial protection measured in CCHFV-Afg09-2990 challenged mice vaccinated with CCHFV-M_10200_. Interestingly, mice vaccinated with CCHFV-M_10200_ had similar levels of anti-G_N_/G_C_ T cell responses following stimulation with either CCHFV-IbAr 10200 or CCHFV-Afg09-2990 peptides, but did not generate significant cellular immunity to the MLD or GP38 regions of CCHFV-Afg09-2990. The response to GP38 was rescued upon CCHFV-M_Afg09_ vaccination, which protected 100% of the animals against CCHFV-Afg09-2990 challenge. Likewise, anti-CCHFV-IbAr 10200 GP38 titers trended lower in CCHFV-M_Afg09_ vaccinated mice and protection against heterologous viral strains can be limited following mAb-13G8 treatment, but heterologous protection may be influenced by the route of treatment, timing, and regions of sequence divergence^17,21^. Most notably, our GP38 DNA vaccine yielded 20% protection, a result that coincided with the two mice with the highest anti-GP38 antibody levels. The high genetic diversity of GP38 between different CCHFV strains may explain the decrease in protection, as subtle differences in the GP38 amino acid sequences can significantly affect both arms of the adaptive immune response (Supplementary Fig. 5). Small changes in amino acid sequence or secondary structures can limit antibody affinity, reducing the effectiveness of neutralizing and non-neutralizing antibodies. Similarly, changes in the amino acid sequence may alter major histocompatibility complex (MHC) I or MHC II antigen presentation, impairing the stimulation of CD4^+^ and/or CD8^+^ effector lymphocytes. GP38 has at least three non-overlapping epitopes, so multiple regions may influence vaccine efficacy^17^. These results suggest that anti-GP38 immunity is critical for a positive challenge outcome, but further studies examining how GP38 genetic diversity impacts CCHFV infection are required.

The mechanism for how anti-GP38 immunity contributes to protection is still under investigation. CCHFV infection frequently results in severe hepatic injury^6,22,23^, but treatment with an anti-GP38 monoclonal antibody prevents liver injury in murine models^17^. This suggests that GP38 may be a viable target for vaccine development, but a GP38 vaccine will require optimization as evidenced by our cross-protection and GP38 vaccine studies. The addition of the MLD may improve efficacy as the MLD elicited a potent effector T cell response, although the MLD is heavily glycosylated and highly variable, complicating its use as a target. Another simple approach may be the addition of a large carrier molecule to GP38 to improve antigen uptake. Alternatively, vaccines expressing the full-length M-segment, such as CCHFV-M_10200_ or CCHFV-M_Afg09_, can be designed to express a GP38 consensus sequence that may provide immunity to divergent CCHFV strains.

In summary, here we show that a DNA vaccine expressing only the M-segment of CCHFV can provide complete protection from homologous challenge, but only partial protection from heterologous challenge. Our data suggest that the diminished protection is at least partially attributable to the genetic diversity of the GP38 region. Future studies to improve anti-GP38 immune response are underway and will provide insight into CCHF’s correlates of protection. In addition, we have yet to explore the durability of protection afforded by CCHFV-M_10200_ or CCHFV-M_Afg09_. As antibody titers did not plateau by one month post-vaccination, it is reasonable to assume that mice will be protected from the challenge for at least several more months. However, this remains to be investigated experimentally. It is also necessary to determine if our M-segment based vaccine confers protective immunity to nonhuman primates.

## Methods

### Ethics statement

Research was conducted under an IACUC approved protocol in compliance with the Animal Welfare Act, PHS Policy, and other Federal statutes and regulations relating to animals and experiments involving animals. The facility where this research was conducted is accredited by the AAALAC, International and adheres to principles stated in the Guide for the Care and Use of Laboratory Animals, National Research Council, 2011^24^. Humane endpoints were used during these studies, and mice that were moribund, according to an endpoint score sheet, were humanely euthanized. Mice were euthanized by CO_2_ exposure using compressed CO_2_ gas followed by cervical dislocation. However, even with multiple observations per day, some animals died as a direct result of the infection.

### Virus production

CCHFV-IbAr 10200 virus was passaged nine times in suckling mouse brain and then propagated three times in Hep G2 cells. The virus was collected from clarified cell culture supernatants and stored at −80 °C. CCHFV-Afg09-2990 virus was derived from a fatal human case in a U.S. soldier stationed in Afghanistan in 2009. CCHFV-Afg09-2990 was passaged three times in Vero cells and then propagated twice in Huh-7 cells (Bernhard Nocht Institute)^25^. Harvested virus was collected from clarified cell culture supernatants and stored at −80 °C. All CCHFV work was performed in BSL-4 containment.

### CCHFV DNA vaccine construction

The M-segment ORF of strain CCHFV-IbAr 10200 (Accession # AAA86616) was optimized by GeneArt for human codon usage and deletion of known motifs that are detrimental to mRNA stability or expression. The optimized gene was de novo synthesized and cloned into pCAGGS. The codon-optimized M-segment ORF was subcloned into the mammalian expression vector pWRG7077 at the NotI sites to create the optimized CCHFV-M_10200_ DNA vaccine^26^. For the CCHFV-M_Afg09_ DNA vaccine, The M-segment ORF of strain CCHFV-Afg09-2990 (Accession # HM452306) was optimized by ATUM Inc. and subcloned into pWRG7077. All nucleotide sequences were confirmed prior to vaccination.

### Flow cytometry

293T cells were propagated in 24-well tissue culture plates (Corning). CCHFV-M_Afg09_ was transfected into 293T cells in a dilution series of 10, 25, 50, 100, and 200 ng in duplicate using FuGENE 6 (Promega) according to manufacturer’s directions. Transfected cells were incubated for 48 h prior to flow cytometry analysis. To detect the intracellular CCHFV glycoprotein, cells were permeabilized with Perm/Wash buffer (BD Biosciences) according to the manufacturer’s instructions. The permeabilized cells were incubated with 5 μg/ml of anti-CCHFV G_C_ mouse monoclonal antibody 11E7 (USAMRIID) in Perm/Wash buffer. Alexa Fluor 488-conjugated goat anti-rabbit IgG (Life Technologies) was diluted 1:200 and incubated with the cells. The cells were then analyzed on a FACSCalibur flow cytometer (BD Biosciences). Cells staining positive for intracellular glycoprotein are shown as a percentage of total cells per 10,000 events.

### Western blot

293T cells in 6-well plates were transfected with 2 µg of CCHFV-M_Afg09_ or mock for 48 h. Following transfection, the cells were lysed with 1X Protein Loading Buffer (LI-COR). The cell lysates were probe sonicated for 15–20 s. For detection of G_N,_ aliquots were mixed 9:1 with 2-mercaptoethanol (Sigma) and heated at 70 °C for 10 min. Additionally, for detection of G_C_ aliquots were prepared without 2-mercaptoethanol and heated at 55 °C for 10 min. Proteins were separated by SDS-PAGE in 10% Bis-Tris gels (NuPAGE) and transferred to polyvinylidene difluoride membranes (Invitrogen). The membranes were blocked with 5% milk in phosphate–buffered Saline (PBS, LI-COR). Blots of non-reduced proteins were probed for G_C_ with 36 μg/ml of mouse monoclonal antibody 11E7 (USAMRIID) and reduced proteins for G_N_ with 1:10,000 rabbit polyclonal anti-CCHFV G_N_ antibody (a generous gift from Ali Mirazimi, Karolinska Institute) prepared in 5% Milk in PBS (Sigma) supplemented with 0.2% Tween-20 (PBST, Sigma) and incubated at 4 °C overnight. The membranes were washed 3 times with PBST and incubated with IR680-conjugated anti-rabbit or IR800-conjugated anti-mouse secondary antibodies (LI-COR) diluted in 5% Milk in PBST at ambient temperature for 1 h. The membranes were washed an additional 3 times with PBST and imaged using an Odyssey CLx imaging system (LI-COR). All Western blots were derived from the same cellular lysates.

### DNA vaccination and viral challenge in mice

Groups of 10 C57BL/6 mice (The Jackson Laboratory) were vaccinated three times at 3-week intervals with 50 µg of the pWRG7077 DNA vaccine plasmid expressing the codon-optimized M-segment from either CCHFV-IbAr 10200 (CCHFV-M_10200_) or CCHFV-Afg09-2990 (CCHFV-M_Afg09_) by IM-EP as previously described^8^. Control groups of 10 C57BL/6 mice (The Jackson Laboratory) were vaccinated concurrently by IM-EP with pWRG7077 empty vector. For IM-EP delivery, mice were anesthetized and then vaccinated in the tibialis anterior muscle with 20 μl of DNA solution using a 3/10 cm3 U-100 insulin syringe inserted into the center of an Ichor Medical Systems TriGrid electrode array (Ichor Medical Systems) with 2.5 mm electrode spacing. Injection of DNA was followed immediately by electrical stimulation at an amplitude of 250 V/cm, and the total duration was 40 ms over a 400 ms interval. Sera were collected prior to vaccination on days 0, 21, and 42 by submandibular bleed. A cohort of mice was euthanized on day 49 for T cell analysis. The remainder of the mice were observed until day 63, when sera were harvested for antibody analysis. Mice were subsequently challenged on day 72. For the challenge, all mice were treated by the IP route with mAb-5A3 (Leinco Technologies Inc.) 24 h prior to (2.0 mg) and 24 h after (0.5 mg) CCHFV challenge. IS C57BL/6 mice were challenged with 100 PFU of CCHFV strain CCHFV-IbAr 10200 or 100 PFU of CCHFV-Afg09-2990 by the IP route. The mice were monitored daily for weight changes, clinical score, and survival. Twenty-six days following challenge, the surviving mice were euthanized by exsanguination under deep anesthesia.

### CCHFV Cell Lysate

T150 flasks with HEK293T cells (ATCC) at 70-80% density were transfected with 15 µg of CCHFV-M_10200_ or CCHFV-M_Afg09_ using Fugene 6 (Promega) according to manufacturer’s instructions. Cells were incubated for 48 h at 37 °C in 5% CO_2_ prior to being lifted by pipetting in media. Cell-laden media were then pelleted at 1,155 x g for 5 min at 4 °C. Supernatant was discarded and cells were washed two times sequentially with cold PBS (Gibco Life Technologies Corp). After final pelleting, the PBS was discarded and cells were lysed with 3 ml of lysis buffer per flask equivalent. Lysis buffer was as follows: 20 mM HEPES (Sigma-Aldrich), 110 mM Potassium Acetate (Sigma-Aldrich), and 2 mM, Magnesium Chloride (Sigma-Aldrich) supplemented with 1% Tween-20 (Sigma-Aldrich) and protease inhibitor tablets (Sigma-Aldrich). Cells were vortexed for 30 s and then lysed on a rocking platform overnight at 4 °C. Debris was cleared at 16,100 × *g* for 10 min at room temperature. Cleared lysate was then stored in single-use aliquots at −80 °C until use in ELISA.

### CCHFV Cell Lysate ELISA

For cell lysate ELISA, High Bind ELISA plates (Corning) were coated overnight at 4 °C with a 1:4 dilution of CCHFV cell lysates diluted in PBS. The following day, plates were washed with PBS containing 0.05% Tween-20 (PBST) (Sigma-Aldrich) and then blocked with PBST containing 3% goat serum (Sigma-Aldrich) and 3% skim milk (BD Biosciences) for 1 h at 37 °C. Plates were washed with PBST again, prior to being loaded with two-fold serial dilutions of mouse sera in duplicate (dilution range 1:200 to 1:25,600). Sera were diluted in blocking buffer. Plates were incubated at ambient temperature for 1 h prior to being washed with PBST, and then incubated with a 1:1000 dilution of horseradish peroxidase (HRP) conjugated goat anti-mouse (SeraCare Life Sciences) in PBST for 1 h at ambient temperature. Plates were washed with PBST again and then developed with TMB substrate (SeraCare Life Sciences). Absorbance at the 450 nm wavelength was detected with a Tecan M1000 microplate reader (Tecan Group Ltd). Pooled naïve sera collected prior to vaccination were used as an internal control for each assay group. A plate cutoff value was determined based on the average absorbance of the naïve control starting dilution plus 3 standard deviations. Only sample dilutions whose average were above this cut-off were registered as a positive signal. Additional analysis was carried out using GraphPad Prism 6 (GraphPad Software).

### T-cell ELISPOT

Mouse T cell ELISPOT reagents were obtained from Mabtech (Mabtech). Antigen-specific IFN-γ^+^ and IL-2^+^ T cells were quantified per manufactures instructions. Positive control wells were stimulated with 10 ng/ml PMA (Sigma-Aldrich) and 500 ng/ml ionomycin (Sigma-Aldrich). Test splenocyte wells were stimulated with the appropriate peptides at a concentration of 2.5 µg/ml. Cells were incubated for 20 h at 37 °C in 5% CO_2_. Positive spots were visualized on a CTL Imager and counting was performed with Immunospot software (Cellular Technology Ltd.). Splenocytes from vaccinated mice were stimulated with pooled 15-mer peptides (9 pools of 17 peptides and 1 pool of 15 peptides) containing a 5-base overlap spanning either the CCHFV-IbAr 10200 or CCHFV-Afg09-2990 M-segment open reading frames (Mimotopes).

### Cloning

All GP38 constructs were produced through de novo synthesis (Genewiz). tPA-GP38 strain CCHFV-IbAr 10200 (NC_005300) was produced by the addition of the tPA secretion signal (MDAMKRGLCCVLLLCGAVFVSPS). Genes were cloned into the NotI and BglII sites of the pWRG7077 vector and verified by sequence analysis. For the histidine-tagged version of tPA-GP38 from strain CCHFV-IbAr 10200, six histidine residues were added to the C-terminal domain of the protein by de novo synthesis and cloned into the HindIII and XhoI site of pBFksr-HCacc-MCS, which contains a cytomegalovirus promotor (Biofactora).

A modified M-segment lacking the mucin and GP38 regions was produced by polymerase chain reaction (PCR). ΔMUCΔGP38 was produced using the forward primer 5′-ATCGCTGGGCTCCTCGCTGTGGCTGCCGTGGGTCTC-3′ and reverse primer 3′-GAGACCCACGGCAGCCACAGCGAGGAGCCCAGCGAT-5′, which removed nucleotide regions 120 to 1545. The ΔMUCΔGP38 construct retained the signal sequences 1 to 117. All PCR reactions were performed using the Phusion polymerase (Invitrogen). Following PCR, fragments were digested with NotI and BglII and ligated into the pWRG7077 vector. Sequence analysis was used to verify that the changes had been successfully incorporated into the gene.

### GP38 purification

Production of recombinant CCHFV-IbAr 10200 GP38his was accomplished by transient transfection of HEK293T cells (American Type Culture Collection) with the tPA-GP38his plasmid using FuGENE 6 (Promega) according to the manufacturer’s instructions as previously described^17^.

### GP38 ELISA

500 ng per well of purified GP38, diluted in 0.1 M carbonate buffer (pH 9.6), was plated on a high-binding 96-well plate (Corning) and incubated overnight at 4 °C. Plates were blocked for 2 h in blocking buffer (PBST containing 5% milk) at 37 °C. Plates were washed four times in PBST and incubated with mouse sera diluted in blocking buffer overnight at 4 °C (dilution range 1:200 to 1:25,600). Plates were washed four times in PBST and incubated with anti-mouse IgG conjugated to horseradish peroxidase diluted 1:1000 (Sigma-Aldrich) for 1 h at 37 °C. Plates were washed again four times in PBST, and 50 µl of ABTS microwell peroxidase 1-component (KPL) was added to each well. Reactions were stopped by adding 50 µl of ABTS stop solution (KPL). The optical density (OD) at 405 nm was read on a Tecan microplate reader (Tecan Group Ltd.).

### MAGPIX cytokine assay

Murine cytokines/chemokines were assayed using a Luminex MAGPIX-based magnetic bead kit (EMD Millipore). Twenty-five cytokines (G-CSF, GM-CSF, IFN-γ, IL-1α, IL-1β, IL-2, IL-4, IL-5, IL-6, IL-7, IL-9, IL-10, IL-12 (p40), IL-12 (p70), IL-13, IL-15, IL-17, IP-10, KC, MCP-1, MIP-1α, MIP-1β, MIP-2, RANTES, TNFα) were analyzed. Assay plates were prepared as per the manufacturer’s instructions. Briefly, 25 μl of prepared standards and controls were added to the appropriate wells of a 96-well round bottom plate. Next, 25 μl of MAGPIX assay buffer was added to background wells and all sample wells. Sera samples, prepared in duplicate, were diluted 1:5 in MAGPIX serum matrix diluent with 25 μl of this preparation added to the appropriate wells. The serum matrix diluent was also added to the background, standard, and controls, 25 μl per well. Pre-mixed magnetic cytokine/chemokine detection beads were vortexed and added to all wells, 25 μl per well. The plates were sealed and covered with foil to protect the contents from light and allowed to incubate on a digital plate shaker (IKA) overnight at 4 °C. Following two washes with 200 μl per well of the MAGPIX sodium dodecyl sulfate (SDS) wash buffer, 25 μl of detection antibodies were added to all wells and incubated on a digital plate shaker for 1 h at room temperature. A streptavidin-phycoerythrin solution was then added to all wells, 25 μl per well, and incubated on a digital plate shaker for 30 min at room temperature. Plates were washed twice with 200 μl per well of SDS buffer. The premixed beads were re-suspended in 150 μl of Bio-Plex MAGPIX Drive Fluid (Bio-Rad), and placed on a digital plate shaker for 5 min at room temperature. The plates were assayed on the MAGPIX instrument using the Millipore xPONENT software (Luminex Corporation). The mean fluorescent intensity (MFI) for each sample was captured and analyzed using a 5-parameter logistic standard curve corrected for background. Sera dilutions were factored into the final data output.

### Statistics

All data analysis was conducted with GraphPad Prism v8.3.1 for Windows. Data are presented as the mean of individual mice±the standard deviation (SD). Vaccine immunogenicity statistical analysis was performed using Student’s *t* test, a one-way ANOVA followed by a Tukey post-test, or a two-way ANOVA with Sidak’s multiple comparison as indicated. Kaplan–Meier survival curve analysis using a log rank test was performed to determine *p* value significance of vaccinated groups surviving lethal challenge compared to the control group.

### Reporting summary

Further information on experimental design is available in the Nature Research Reporting Summary linked to this paper.

## Supplementary information

Supplementary Information

Reporting Summary

## Data Availability

The data that support the findings of this study are available from the authors on reasonable request pending approval from all relevant government institutions.
