# Supplementary material for: A CCHFV DNA vaccine protects against heterologous challenge and establishes GP38 as immunorelevant in mice
Source: NPJ Vaccines. 2021 Mar 2;6:31. doi: 10.1038/s41541-021-00293-9 (PMC7925670; doi:10.1038/s41541-021-00293-9)
Supplement: Supplementary file 1 — Supplementary Information [file 41541_2021_293_MOESM1_ESM.pdf]

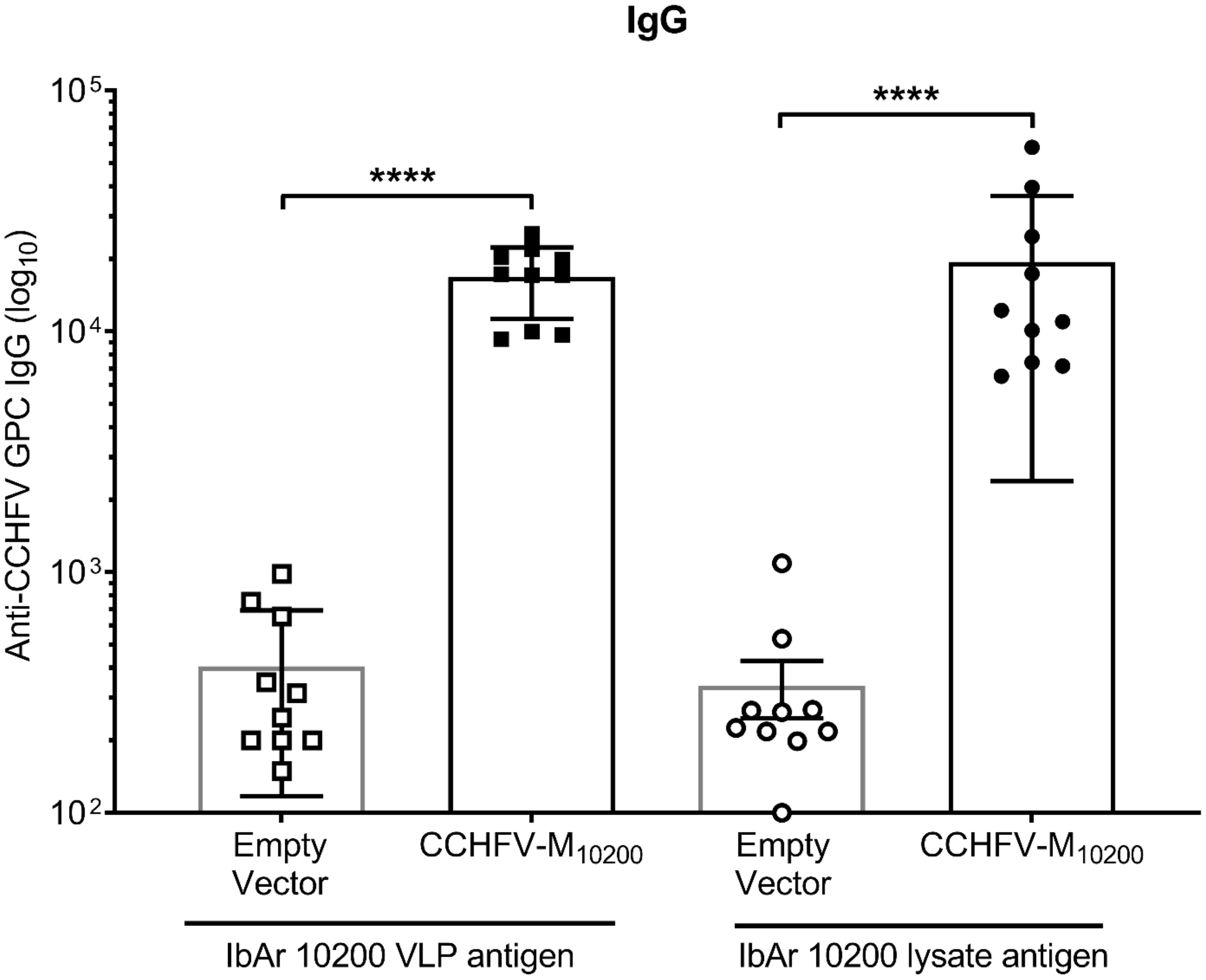

Supplementary Fig. 1: Comparison of CCHFV GPC-specific antibody titers in CCHFV-M_{10200} vaccinated mice by ELISA coated with either secreted $CCHF_{VLP}$ or CCHFV-IbAr 10200 $CCHF_{VLP}$ transfected cell lysates.

Groups of 10 female C57BL/6 mice were vaccinated with 50 µg CCHFV-M_{10200} or empty vector on days 0, 21, and 42 by IM-EP. Sera antibody responses were analyzed 21 days post third vaccination. ELISA plates were coated with either secreted $CCHF_{VLP}$ or CCHFV-IbAr 10200 $CCHF_{VLP}$ transfected cell lysates as indicated. Data are the group mean averages +/- SD. ****$p < 0.0001$. p values were determined by one-way ANOVA with Tukey's post hoc test with a 95% confidence interval.

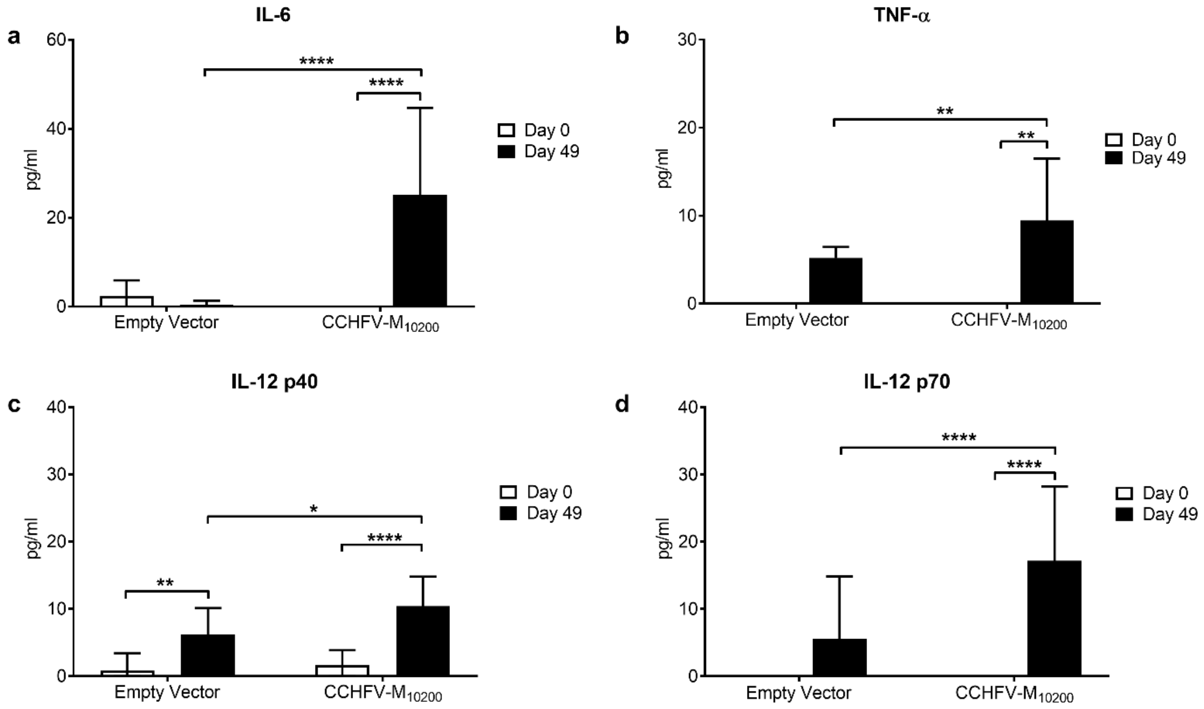

Supplementary Fig. 2: CCHFV-M$_{10200}$ vaccination stimulates T$_h$1 and T$_h$2 cytokine signaling.

10 female C57BL/6 mice were vaccinated with 50 µg CCHFV-M$_{10200}$ or empty vector on days 0, 21, and 42 by IM-EP, and then euthanized on day 49. Sera were collected at the time of euthanasia and cytokine signaling was quantified by Luminex assay. Shown are significant differences in cytokines after vaccination. Data are the group mean averages +/- SD. *p <0.05; **p <0.01; ****p <0.0001. p values were determined by one-way ANOVA with Tukey’s post hoc test with a 95% confidence interval.

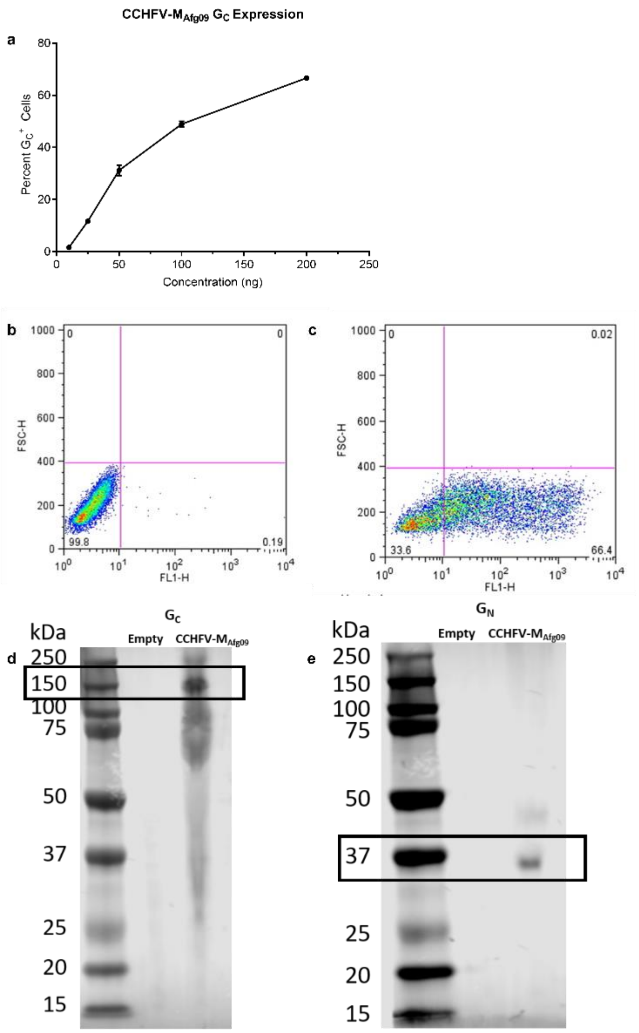

Supplementary Fig. 3: *In vitro* expression of the glycoprotein genes from the CCHFV-M_{Afg09} DNA vaccine plasmid.

a The total (permeabilized cells) of G_C was examined 48 hours after transfection of 293T cells with CCHFV-M_{Afg09} over a dose curve ranging from 10 to 200 ng of plasmid. Figure is representative of 2 independent experiments performed in duplicate. Representative gating strategy for G_C expression in 293T cells transfected with 200 ng of **b** empty vector or **c** CCHFV-M_{Afg09}. *In vitro* expression by Western blot of **d** G_C (75 kDa) and **e** G_N (37 kDa) in 293T cells 48 hours after transfection of 250 ng of CCHFV-M_{Afg09} or empty vector. Note: As G_C is non-reduced, it runs larger than 75 kDa.

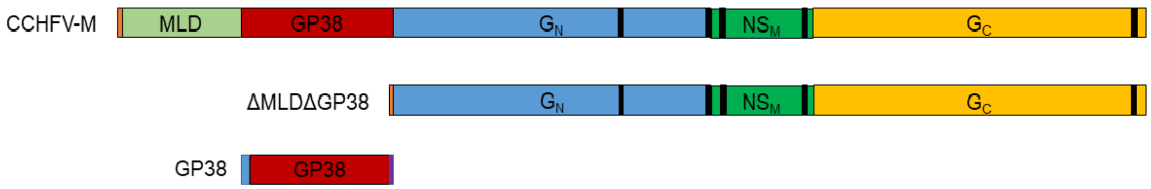

Supplementary Fig. 4: Schematic representations of truncated CCHFV DNA vaccines.

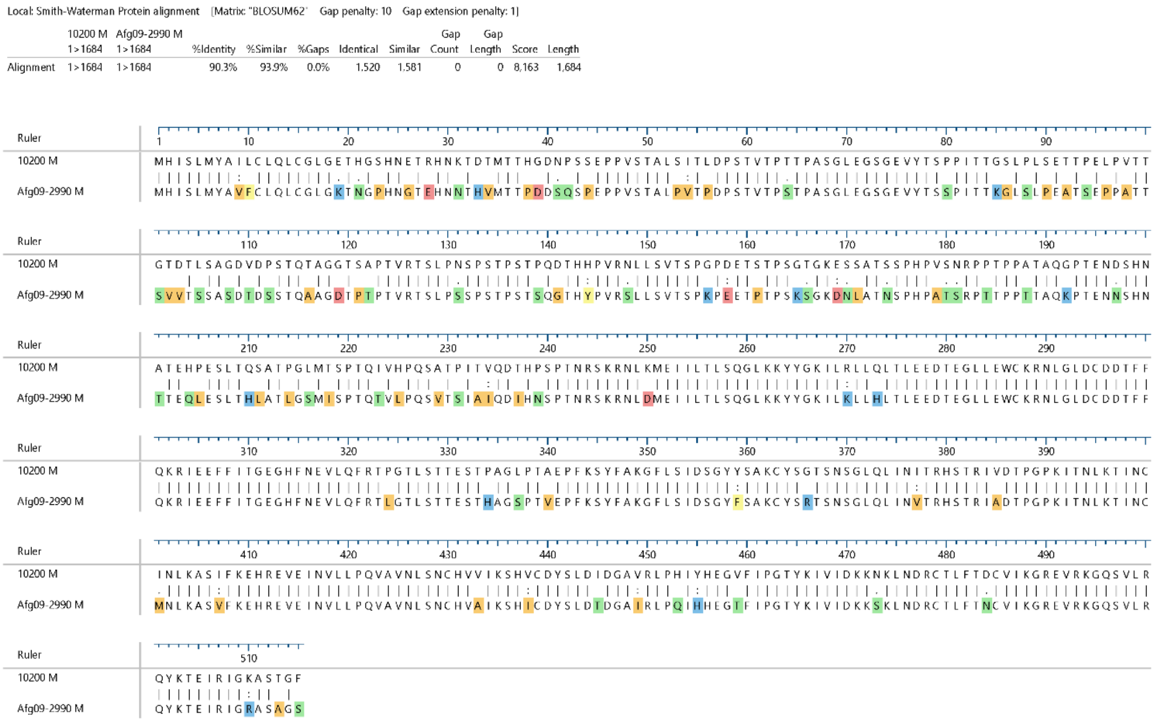

Supplementary Fig. 5: Sequence alignment of MLD and GP38.

Smith-Waterman protein sequence alignment of the MLD and GP38 domains of CCHFV-IbAr 10200 and CCHFV-Afg09-2990.
