# Supplementary material for: A CCHFV DNA vaccine protects against heterologous challenge and establishes GP38 as immunorelevant in mice
Source: NPJ Vaccines. 2021 Mar 2;6:31. doi: 10.1038/s41541-021-00293-9 (PMC7925670; doi:10.1038/s41541-021-00293-9)
Supplement: Supplementary file 2 — Reporting Summary [file 41541_2021_293_MOESM2_ESM.pdf]

nature research

Corresponding author(s): John J. Suschak and Aura R. Garrison

Last updated by author(s): Jan 8, 2021

Reporting Summary

Nature Research wishes to improve the reproducibility of the work that we publish. This form provides structure for consistency and transparency in reporting. For further information on Nature Research policies, see our Editorial Policies and the Editorial Policy Checklist.

Statistics

For all statistical analyses, confirm that the following items are present in the figure legend, table legend, main text, or Methods section.

n/a	Confirmed	
☐	☒	The exact sample size (n) for each experimental group/condition, given as a discrete number and unit of measurement
☐	☒	A statement on whether measurements were taken from distinct samples or whether the same sample was measured repeatedly
☐	☒	The statistical test(s) used AND whether they are one- or two-sided<br>*Only common tests should be described solely by name; describe more complex techniques in the Methods section.*
☒	☐	A description of all covariates tested
☒	☐	A description of any assumptions or corrections, such as tests of normality and adjustment for multiple comparisons
☐	☒	A full description of the statistical parameters including central tendency (e.g. means) or other basic estimates (e.g. regression coefficient) AND variation (e.g. standard deviation) or associated estimates of uncertainty (e.g. confidence intervals)
☐	☒	For null hypothesis testing, the test statistic (e.g. F, t, r) with confidence intervals, effect sizes, degrees of freedom and P value noted<br>*Give P values as exact values whenever suitable.*
☒	☐	For Bayesian analysis, information on the choice of priors and Markov chain Monte Carlo settings
☒	☐	For hierarchical and complex designs, identification of the appropriate level for tests and full reporting of outcomes
☒	☐	Estimates of effect sizes (e.g. Cohen's d, Pearson's r), indicating how they were calculated

Our web collection on statistics for biologists contains articles on many of the points above.

Software and code

Policy information about availability of computer code

Data collection	Not applicable.
Data analysis	Graphpad Prism was used for data analysis.

For manuscripts utilizing custom algorithms or software that are central to the research but not yet described in published literature, software must be made available to editors and reviewers. We strongly encourage code deposition in a community repository (e.g. GitHub). See the Nature Research guidelines for submitting code & software for further information.

Data

Policy information about availability of data

All manuscripts must include a data availability statement. This statement should provide the following information, where applicable:

- Accession codes, unique identifiers, or web links for publicly available datasets
- A list of figures that have associated raw data
- A description of any restrictions on data availability

The datasets generated during and/or analysed during the current study are available from the corresponding author on reasonable request.

Field-specific reporting

Please select the one below that is the best fit for your research. If you are not sure, read the appropriate sections before making your selection.

☒ Life sciences ☐ Behavioural & social sciences ☐ Ecological, evolutionary & environmental sciences

For a reference copy of the document with all sections, see nature.com/documents/nr-reporting-summary-flat.pdf

Life sciences study design

All studies must disclose on these points even when the disclosure is negative.

Sample size	All sample sizes were determined to be significant by an in-house statistician.
Data exclusions	No data was excluded from this report.
Replication	In vitro studies were repeated in multiplelndependent studies. In vivo studies were accomplished with a statistically significant n value for each group to ensure reproducibility.
Randomization	All in vivo study groups were assigned at random. Individual mice were randomly assigned to a housing pan by independent veterinary staff with no input by the study team.
Blinding	The study team was not blinded to the grouping. The lead investigator performed all vaccinations, challenges, and assays included in this report.

Reporting for specific materials, systems and methods

We require information from authors about some types of materials, experimental systems and methods used in many studies. Here, indicate whether each material, system or method listed is relevant to your study. If you are not sure if a list item applies to your research, read the appropriate section before selecting a response.

Materials & experimental systems

n/a	Involved in the study	
☐	☒	Antibodies
☐	☒	Eukaryotic cell lines
☒	☐	Palaeontology and archaeology
☐	☒	Animals and other organisms
☒	☐	Human research participants
☒	☐	Clinical data
☒	☐	Dual use research of concern

Methods

n/a	Involved in the study	
☒	☐	ChIP-seq
☒	☐	Flow cytometry
☒	☐	MRI-based neuroimaging

Antibodies

Antibodies used	Flow cytometry gene expression data was accomplished by use of in-house monoclonal antibodies (11E7). These antibodies may be available by reasonable request to the corresponding author.
Validation	These antibodies have previously been used to characterize the reported genes. Previous reports are available.

Eukaryotic cell lines

Policy information about cell lines

Cell line source(s)	293T cell lines are maintained in house
Authentication	The 293T cells used are authenticated and maintained by the Cell Culture division of USAMRIID.
Mycoplasma contamination	All cell lines used are regularly tested by the Cell Culture division of USAMRIID and were negative for mycoplasma contamination.
Commonly misidentified lines (See ICLAC register)	No such cell lines were used.

Animals and other organisms

Policy information about studies involving animals; ARRIVE guidelines recommended for reporting animal research

Laboratory animals	Female C57BL/6 mice, aged 6-8 weeks, were used in these studies.
Wild animals	Not applicable.
Field-collected samples	Not applicable.
Ethics oversight	Research was conducted under an IACUC approved protocol in compliance with the Animal Welfare Act, PHS Policy, and other Federal statutes and regulations relating to animals and experiments involving animals. The facility where this research was conducted is accredited by the Association for Assessment and Accreditation of Laboratory Animal Care, International and adheres to principles stated in the Guide for the Care and Use of Laboratory Animals, National Research Council, 2011

Note that full information on the approval of the study protocol must also be provided in the manuscript.
